# Phase 2 study of cabazitaxel as second-line treatment in patients with HER2-negative metastatic breast cancer previously treated with taxanes—a Hellenic Cooperative Oncology Group (HeCOG) Trial

**DOI:** 10.1038/s41416-020-0909-4

**Published:** 2020-06-03

**Authors:** Angelos Koutras, Flora Zagouri, Georgia-Angeliki Koliou, Elizabeth Psoma, Ioannis Chryssogonidis, Georgios Lazaridis, Dimitrios Tryfonopoulos, Athanasios Kotsakis, Eleni Res, Nikolaos K. Kentepozidis, Evangelia Razis, Amanda Psyrri, Georgios Koumakis, Haralabos P. Kalofonos, Meletios A. Dimopoulos, George Fountzilas

**Affiliations:** 1 Division of Oncology, Department of Medicine, University Hospital, University of Patras Medical School, Patras, Greece; 2 Department of Clinical Therapeutics, Alexandra Hospital, National and Kapodistrian University of Athens School of Medicine, Athens, Greece; 3 Section of Biostatistics, Hellenic Cooperative Oncology Group, Data Office, Athens, Greece; 4 Department of Radiology, AHEPA Hospital, Aristotle University of Thessaloniki, School of Health Sciences, Faculty of Medicine, Thessaloniki, Greece; 5 Department of Medical Oncology, Papageorgiou Hospital, Aristotle University of Thessaloniki, School of Health Sciences, Faculty of Medicine, Thessaloniki, Greece; 6 Second Department of Internal Medicine, Agios Savvas Cancer Hospital, Athens, Greece; 7 Department of Medical Oncology, University General Hospital of Heraklion Crete, Heraklion, Greece; 8 Third Department of Medical Oncology, Agii Anargiri Cancer Hospital, Athens, Greece; 9 Department of Medical Oncology, 251 Airforce General Hospital, Athens, Greece; 10 Third Department of Medical Oncology, Hygeia Hospital, Athens, Greece; 11 Section of Medical Oncology, Department of Internal Medicine, Attikon University Hospital, National and Kapodistrian University of Athens School of Medicine, Athens, Greece; 12 Aristotle University of Thessaloniki, Thessaloniki, Greece; 13 German Oncology Center, Limassol, Cyprus

**Keywords:** Breast cancer, Outcomes research

## Abstract

**Background:**

Cabazitaxel is a novel taxane that might be active in breast cancer resistant to first-generation taxanes.

**Methods:**

The purpose of the current multicentre phase II trial was to evaluate the activity and safety of cabazitaxel, as second-line treatment, in patients with human epidermal growth factor receptor 2 (HER2)-negative metastatic breast cancer (MBC) previously treated with taxanes. The primary endpoint was objective response rate (ORR).

**Results:**

Eighty-four patients were enrolled between October 2012 and November 2016. Taxane resistance to previous treatment was detected in 43 cases. The ORR was 22.6% in the intent-to-treat population, 23.3% in taxane-resistant and 20.5% in taxane-non-resistant cases. At a median follow-up of 39.6 months, the median progression-free survival and overall survival were 3.7 months (95% CI 2.2–4.4) and 15.2 months (95% CI 11.3–19.4), respectively. Regarding toxicity, grade 3–4 neutropenia was reported in 22.6% and febrile neutropenia in 6% of the patients, respectively. Two fatal events (one febrile neutropenia and one sepsis) were reported as being related to study treatment.

**Conclusions:**

This phase II trial suggests that cabazitaxel is active as second-line treatment in taxane-pretreated patients with HER2-negative MBC, with manageable toxicity.

## Background

Metastatic breast cancer (MBC) continues to be an incurable disease in the majority of cases. In this clinical setting, the role of systemic treatment is to palliate symptoms, maintain quality of life and prolong patient survival. In patients with hormone receptor-positive, human epidermal growth factor receptor 2 (HER2)-negative MBC, international guidelines support the administration of endocrine therapy (with or without targeted agents) in the majority of cases, excluding only those with visceral crisis or endocrine resistance.^1^ However, chemotherapy remains the standard approach in endocrine-refractory or aggressive cases.

The application of anthracyclines and taxanes in the first-line treatment has been associated with an improvement in the survival of patients with advanced breast cancer.^2^ When progression of the disease develops following first-line chemotherapy, further lines of systemic therapy may be administered.^3^ However, in patients with MBC resistant to anthracyclines and/or taxanes, well documented active treatments are limited. Furthermore, with the extensive use of anthracyclines and taxanes in chemotherapy regimens administered as adjuvant therapy in early breast cancer, the development of active options in patients with MBC, particularly those pretreated with taxanes, is considered important.

Cabazitaxel (XRP6258) is a recent taxane, showing activity in vitro and in vivo in cell lines and tumours resistant to docetaxel and paclitaxel. Cabazitaxel is also able to penetrate the blood–brain barrier.^4^ The greater activity of cabazitaxel as compared to other taxanes is attributed to a faster uptake and longer retention by tumour cells.^5^

Weekly and every 3-week schedules of administration of cabazitaxel were evaluated in phase I studies. In one trial, cabazitaxel was administered on a weekly regimen as a 1-h i.v. infusion on days 1, 8, 15 and 22 every 5 weeks, in patients with advanced solid tumours.^6^

Two additional trials (TED6188 and TED6190) evaluated cabazitaxel given as a 1-h i.v. infusion every 3 weeks. The dose-limiting toxicity of cabazitaxel was neutropenia and its infectious complications at the highest dose tested, 30 mg/m² in TED6188 and 25 mg/m² in TED6190.^7,8^

Consequently, the dose levels of 25 mg/m² and 20 mg/m² every 3 weeks were defined as the recommended doses for further clinical development.

A phase III clinical trial has documented the activity of cabazitaxel in patients with castration-resistant metastatic prostate cancer and disease progression during or following previous treatment with docetaxel.^9^ Preclinical and limited clinical data indicate that cabazitaxel might also be effective in patients with breast cancer resistant to first-generation taxanes.^10,11^

We conducted a multicentre, non-randomised Phase 2 trial to assess the activity and safety of cabazitaxel as second-line treatment in patients with HER2-negative MBC, previously treated with taxanes. The primary objective was to evaluate the clinical efficacy of cabazitaxel regarding the objective response rate (ORR). Secondary objectives included duration of response (DOR), progression-free survival (PFS), overall survival (OS) and safety.

## Methods

### Patient selection

Eligible patients were women with HER2-negative metastatic breast adenocarcinoma, who had measurable disease by the Response Criteria in Solid Tumors 1.1 (RECIST 1.1).^12^ All cases were to have received first-line chemotherapy for locally recurrent/metastatic disease. Moreover, eligible patients should have received prior taxane-containing treatment (paclitaxel, docetaxel or nab-paclitaxel), either for advanced disease or as neoadjuvant/adjuvant chemotherapy. Additional eligibility criteria included: age 18–75 years, an Eastern Cooperative Oncology Group performance status (PS) of 0–1, life expectancy of at least 12 weeks, adequate organ function including haemoglobin ≥ 9.0 g/dL; absolute neutrophil count of ≥1.5 × 10^9^/L; thrombocyte count of ≥100 × 10^9^/L; serum creatinine ≤ 1.5 upper limit of normal (ULN); SGOT (AST), SGPT (ALT) ≤ 2.5 × ULN; alkaline phosphatase ≤ 2.5 × ULN; total bilirubin ≤ ULN. The enrolment of patients with controlled brain metastases was allowed.

Patients who had received more than one line of chemotherapy for locally recurrent/metastatic disease were excluded. Furthermore, patients were not eligible for study enrolment in the following cases: neuropathy CTC grade 2 or greater at baseline; history of severe hypersensitivity reaction (≥grade 3) to docetaxel or polysorbate 80 containing drugs; spinal cord compression or carcinomatous meningitis; any concurrent active malignancy other than non-melanoma skin cancer or in situ carcinoma of the cervix; clinically significant cardiac disease within 6 months from study entry; uncontrolled severe illness or medical condition; any other significant acute or chronic medical or psychiatric condition or abnormal laboratory finding; pregnancy or lactation; concurrent or planned treatment with strong inhibitors or strong inducers of cytochrome P450.

### Treatment protocol

Cabazitaxel (XRP6258) was administered on day 1 of each cycle at a dose of 25 mg/m², as an i.v. 1-h infusion. Cycle length for cabazitaxel was 3 weeks. At least 30 min prior to each administration of cabazitaxel, patients were given i.v. premedication for hypersensitivity prophylaxis including: an anti-histamine, a corticosteroid and an H2 antagonist. Anti-emetic prophylaxis was also recommended. Primary prophylaxis with granulocyte-colony stimulating factor (G-CSF) was recommended in all patients, due to the high incidence of cabazitaxel-associated neutropenia. Treatment was continued until consent withdrawal by the patient, intolerable toxicity or documented disease progression. Patients could not begin a new cycle of treatment unless the neutrophil count was at least 1.5 × 10^9^/L and the platelet count was at least 75 × 10^9^/L, and non-haematological toxicities (except alopecia) had improved. Toxicities required resolution to grade 1 or to baseline before the next cycle of treatment could be administered. A maximum of 2-week delay was allowed between treatment cycles. Patients would come off treatment if treatment had to be delayed by more than 2 weeks. Dose could be reduced for cabazitaxel when necessary. The dose, which had been reduced for toxicity, should not be re-escalated. Only one dose reduction (20 mg/m²) was allowed per patient. If a second dose reduction was required, the patient had to discontinue study treatment.

### Clinical assessment

Measurable disease was assessed by imaging studies, preferably CT scans, during the duration of the treatment using the RECIST 1.1 criteria. Imaging evaluation included CT scans of the brain, lungs, upper/lower abdomen, as well as bone scans. Patients underwent baseline imaging during the 2 weeks preceding treatment initiation and were rescanned, using the same method, 8, 16 and 24 weeks after the initiation of the treatment and every 3 months thereafter. The radiological assessment of response rate was performed centrally according to the RECIST 1.1 criteria upon completion of the study, after review of the imaging tests by two experienced radiologists (EP, IC). Toxicities were assessed using the NCI Common Terminology Criteria for Adverse Events version 4 at baseline and at the conclusion of each cycle of treatment.

### Statistical analysis

The primary endpoint of the study was to assess the clinical activity of cabazitaxel regarding the ORR. According to the Simon’s optimal two-stage design, assuming that the expected ORR would be at least 25% and the minimum acceptable response rate was 15%, with a type I and II error of 10 and 20%, respectively, a total of 29 patients was required for the first stage of the study. Upon enrolment of the 29th patient, recruitment was halted in order to perform an interim analysis for response assessment. Given the number of responses observed in the first stage, 55 additional patients were enrolled, leading to a total of 84 patients. Secondary endpoints included assessment of PFS, OS, DOR and the safety profile of cabazitaxel. Time to treatment failure (TTF) and time to response (TTR) were also assessed.

Progression-free survival was calculated from the date of study entry to the date of first documented disease progression, death (from any cause) without prior documented progression or last contact (whichever occurred first). Overall survival was calculated from the date of study entry to the date of patient’s death or last contact, while TTF was measured from the date of study entry to the date of treatment discontinuation for any reason, including disease progression, treatment toxicity and death. Alive patients without progression, that did not discontinue treatment due to toxicity or any other reason, were censored at the date of last contact. Time to response was measured from the date of study entry until measurement criteria were first met for a partial or complete response. Duration of response was measured as the time from the date of onset of complete or partial response until the date of progression, death from any cause or last contact, whichever occurred first. Time to event data were analysed using the Kaplan–Meier product limit method and compared across groups with the log-rank test.

The study was conducted on an intent-to-treat (ITT) basis and therefore all enrolled patients were included in the analysis. Analysis of response was conducted separately in the intent-to-treat population as well as in the subgroup of patients with measurable disease who received at least two cycles of cabazitaxel (response evaluable population). In addition, cases with early disease progression (prior to cycle 2) were not excluded from this subgroup of patients. The safety profile of cabazitaxel was assessed in the safety population consisting of patients who received at least one dose of the investigational product.

In an unplanned analysis, we evaluated the efficacy of cabazitaxel in patients with and without taxane-resistant disease. Taxane resistance was defined as: For advanced disease, progressive disease as the best overall response on first-line treatment with a taxane; or, progressive disease within 4 months following discontinuation of first-line treatment with a taxane, after an initial objective response or disease stabilisation. For patients who had received an adjuvant or neoadjuvant taxane-containing regimen, a disease-free interval of ≤12 months from the end of treatment.^10^

Moreover, we evaluated the activity of cabazitaxel in the subgroups of patients with triple-negative and luminal disease.

All tests were two-sided and the significance level was set at 5%. The data cut-off date for the analysis was June 20, 2018. The SAS version 9.3 (SAS Institute) was used for statistical analysis and the R studio version 3.5.0 was used for generation of survival plots.

## Results

### Patient characteristics

Eighty-four patients with a median age of 57.5 years (range, 33.5–74.9) were enrolled between October 2012 and November 2016. Among them, 3 (3.6%) were ineligible (one patient with a neuroendocrine tumour, one without previous taxane treatment and one patient with HER2-positive disease) but received at least two cycles of treatment. Patient and tumour characteristics at study entry are summarised in Table 1. The majority of the patients were postmenopausal (85.7%), with PS 0 (89.3%). Oestrogen receptor (ER)/progesterone receptor (PgR)-positivity was found in 67.9% of cases, while 29.8% of patients had triple-negative tumours. Moreover, taxane resistance was detected in 43 patients (51.2%).

**Table 1:** Patient and tumour characteristics at study entry.

	Total (*N* = 84)
Age	
Median (range)	57.5 (33.5–74.9)

	N (%)
Menopausal status	
Premenopausal	12 (14.3)
Postmenopausal	72 (85.7)
Histological classification	
Invasive ductal	67 (79.8)
Inflammatory	1 (1.2)
Medullary with lymphatic	2 (2.4)
Mucinous	1 (1.2)
Invasive lobular	9 (10.7)
Mixed	2 (2.4)
Other^a^	2 (2.4)
Histology grade	
I	2 (2.4)
II	30 (35.7)
III	46 (54.8)
Unknown	6 (7.1)
Performance status	
0	75 (89.3)
1	9 (10.7)
ER/PgR status	
Positive	57 (67.9)
Negative	26 (31.0)
Unknown	1 (1.2)
Prior chemotherapy exposure	
Adjuvant chemotherapy	57 (67.9)
Anthracycline-containing adjuvant chemotherapy	48 (57.1)
Neoadjuvant chemotherapy^b^	6 (7.1)
First-line chemotherapy	80 (95.2)
Anthracycline-containing first-line chemotherapy	11 (13.1)
Taxane resistance	
Taxane-non-resistant disease	39 (46.4)
Taxane-resistant disease	43 (51.2)
Unknown	2 (2.4)
N of prior lines of hormonal therapy for advanced disease^c^	
0	13 (22.8)
1	22 (38.6)
2	5 (8.8)
3	4 (7.0)
Unknown	3 (5.3)
Site of metastasis	
Liver	52 (61.9)
Lung	28 (33.3)
Bones	32 (38.1)
Brain	2 (2.4)
Axillary LNS	12 (14.3)
LNS	19 (22.6)
Other	22 (26.2)
Unknown	2 (2.4)

*N* number, *ER* oestrogen receptor, *PgR* progesterone receptor, *LNS* lymph nodes.

^a^One patient with a neuroendocrine tumour and one with microinvasions.

^b^Anthracycline-containing.

^c^For patients with hormonal-receptor-positive tumours.

### Drug exposure and compliance

**Fig. 1:**
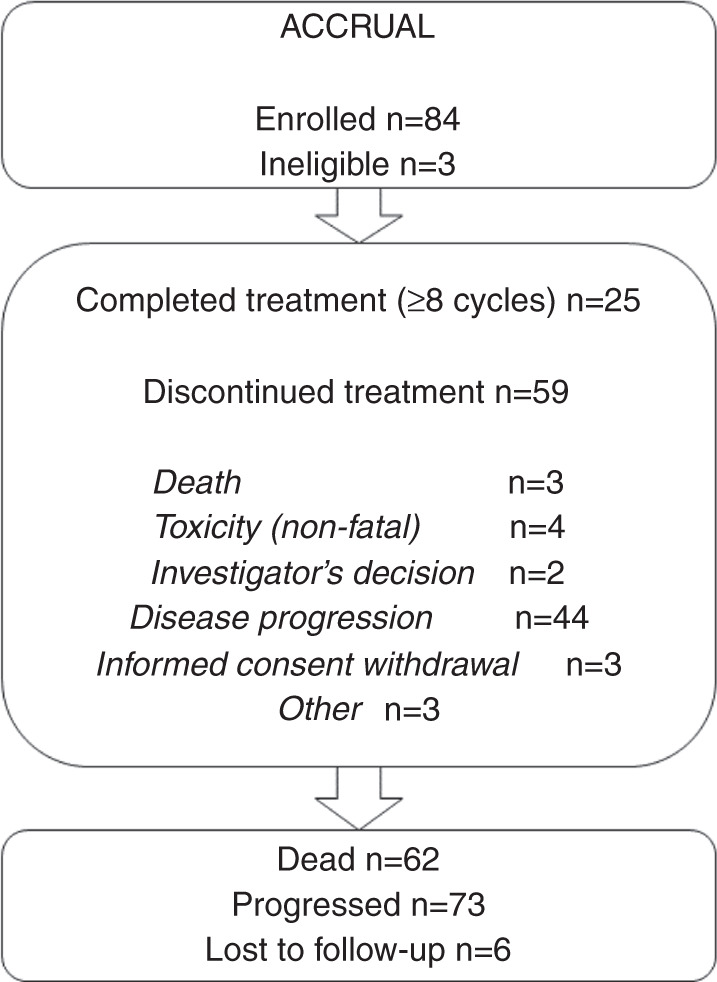
REMARK diagram.

All patients enrolled in the study received at least one cycle of treatment. In total, 499 cycles of cabazitaxel were administered (median 4; range 1–39). The median relative dose intensity was 0.99 (range 0.79–1.49), while dose reduction was required in 13 patients due to adverse events (11 patients) or other reasons (two patients). In nine of them, the dose of cabazitaxel was reduced during the first eight cycles of treatment. In total, 25 patients (29.8%) completed eight cycles of treatment, while the rest of the patients (70.2%) discontinued treatment before the completion of eight cycles. The main reasons for treatment discontinuation were death in three patients (two fatal adverse events), non-fatal adverse events in four patients, investigator’s decision in two patients, disease progression in 44 patients, informed consent withdrawal in three patients and other reasons in three cases (Fig. 1).

### Treatment activity

Among the 84 patients enrolled in the study, objective response was observed in 19 patients (22.6%, 95% CI 14.2–33.1) (one patient with complete and 18 patients with partial response), as assessed by the investigators in the local institutions (Supplementary Table 1). Twenty-six patients presented with stable disease (31.0%), whereas 33 patients (39.3%) had disease progression. Six patients were not evaluated for response due to treatment discontinuation prior to evaluation (four patients), early toxic death (one patient) and early death due to cancer (one patient). Ten of the 43 taxane-resistant patients (23.3%, 95% CI 11.8–38.6) achieved an objective response (one patient with complete response; nine patients with partial response), while objective (partial) response was observed in eight of the 39 patients (20.5%, 95% CI 9.3–36.5) with no resistance to previous taxane treatment. One of the 19 patients with objective response according to the investigators’ assessment in the local hospitals had not received previous taxane treatment. The ORR in patients with triple-negative tumours (*N* = 25) was 20% (95% CI 6.8–40.7%) and among those with luminal disease (*N* = 57) 22.8% (95% CI 12.7–35.8%).

The median time to objective response for the 19 patients was 60 days (range 35.0–128.0), while the median duration of response was 5.6 months (range 0.7–23.2). Taxane-resistant patients (10 cases) achieved an objective response in a median of 64 days (range 36.0–120.0), while the median TTR for the eight taxane-non-resistant patients was 59.5 days (range 37.0–128.0). Taxane-resistant patients had a median duration of response of 5.1 months (range 1.9–23.2), which was shorter than that of non-resistant cases who had a median DOR of 8.6 months (range 1.4–20.4).

**Fig. 2:**
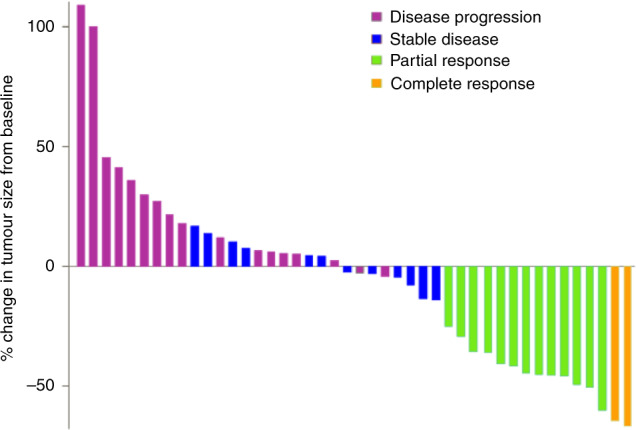
Waterfall plot of responses after central radiological assessment by RECIST 1.1 criteria.

Central radiological review according to RECIST 1.1 criteria was available for 53 cases. Among them, two patients (3.8%) had a complete response, 15 patients (28.3%) achieved a partial response, while stable disease was observed in 15 patients (28.3%) and disease progression in 21 patients (39.6%). A waterfall plot of responses is depicted in Fig. 2. The ORR in the 25 taxane-resistant patients with available central radiological assessment was 32.0% (95% CI 15.0–53.5), while 30.8% (95% CI 14.3–51.8) of the 26 taxane-non-resistant patients with central assessment achieved an objective response. The median time to objective response for the 17 patients was 68 days (range 33.0–174.0), while the median duration of response was 6.7 months (range 1.9–46.5).

**Fig. 3:**
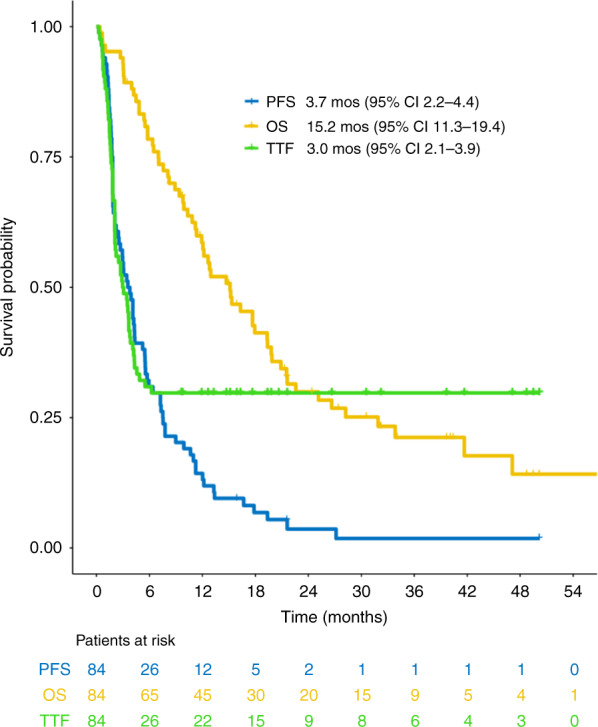
Kaplan–Meier plots with respect to PFS, OS and TTF in the ITT population.

**Fig. 4:**
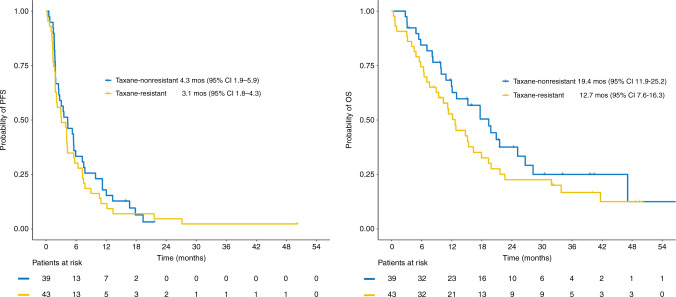
Kaplan–Meier plots with respect to PFS and OS in taxane-resistant and taxane-non-resistant patients.

Within a median follow-up of 39.6 months (95% CI 30.6–48.7), 73 patients (86.9%) had experienced disease progression and 62 patients (73.8%) had died. Sixty patients died from their disease, while in two patients death was attributed to an adverse event. Six patients (7.1%) were lost to follow-up. The median PFS was 3.7 months (95% CI 2.2–4.4) and the median OS was 15.2 months (95% CI 11.3–19.4), while the median TTF was 3.0 months (95% CI 2.1–3.9) (Fig. 3). Taxane-resistant patients presented with numerically shorter PFS [median 3.1 months (95% CI 1.8–4.3) vs. 4.3 months (95% CI 1.9–5.9)] and OS [median 12.7 months (95% CI 7.6–16.3) vs. 19.4 months (95% CI 11.9–25.2)] compared to those who did not develop resistance to previous taxane treatment (Fig. 4). The median TTF for taxane-resistant patients was 2.9 months (95% CI 2.1–4.2), while the median TTF for taxane-non-resistant cases was 3.5 months (95% CI 1.9–4.9).

Among patients with triple-negative disease, the median PFS and OS was 2.1 (95% CI 1.5–4.3) and 13 months (95% CI 9.4–19.3), respectively, while the median TTF was 2.1 months (95% CI 1.6–3.7). In the subgroup of patients with luminal disease, the median PFS, OS and TTF was 4.1 (95% CI 2.6–5.6), 17.7 (95% CI 11.3–21.6) and 3.6 months (95% CI 2.2–4.9), respectively.

### Safety

During the reporting period of the study, 443 events were recorded in 72 patients (85.7%), which were consistent with the already known safety profile of cabazitaxel and other taxanes, as well as the study disease and its complications. Selected grade 1–2 adverse events occurring in more than 10% of patients are depicted in Supplementary Table 2, while Table 2 presents the incidence of grade 3–5 adverse events. Grade 3–4 neutropenia was reported in 22.6% and febrile neutropenia in 6% of patients, respectively. Although severe neutropenic events were observed throughout the study, most of them were manageable and fully recovered. This was probably due to the administration of prophylactic agents and the previous knowledge regarding the safety profile of cabazitaxel. Two fatal events were reported (one febrile neutropenia and one sepsis) that were related to study treatment. The most frequent non-haematological events were fatigue (25 patients; 29.8%), diarrhoea (15 patients; 17.9%) and nausea (10 patients; 11.9%) (all grade 1–3). A total of 29 serious adverse events were reported throughout the study, while there were no unexpected serious adverse events and no new safety signals were revealed.

**Table 2:** Incidence of grade 3, 4 and 5 adverse events.

	Grade 3			Grade 4			Grade 5		
	N of events	N of pts	% of pts	N of events	N of pts	% of pts	N of events	N of pts	% of pts
*Overall*	46	30	35.7	21	15	17.9	2	2	2.4
Haematological									
Febrile neutropenia	3	3	3.6	1	1	1.2	1	1	1.2
Leukopenia	9	9	10.7	3	3	3.6	0	0	0.0
Neutropenia	6	6	7.1	13	13	15.5	0	0	0.0
Lymphopenia	2	2	2.4	0	0	0.0	0	0	0.0
Anemia	2	2	2.4	0	0	0.0	0	0	0.0
Thrombocytopenia	1	1	1.2	2	2	2.4	0	0	0.0
General disorders									
Fatigue	5	5	6.0	0	0	0.0	0	0	0.0
Pain	1	1	1.2	0	0	0.0	0	0	0.0
Infections									
Neutropenic necrotic enterocolitis	0	0	0.0	1	1	1.2	0	0	0.0
Sepsis	0	0	0.0	0	0	0.0	1	1	1.2
Skin infection	1	1	1.2	0	0	0.0	0	0	0.0
Laboratory abnormalities									
Alkaline phosphatase elevation	2	2	2.4	0	0	0.0	0	0	0.0
γGT elevation	2	2	2.4	1	1	1.2	0	0	0.0
LDH elevation	1	1	1.2	0	0	0.0	0	0	0.0
Metabolic abnormalities									
Hypermagnesemia	1	1	1.2	0	0	0.0	0	0	0.0
Hypokalemia	2	2	2.4	0	0	0.0	0	0	0.0
Musculoskeletal symptoms									
Bone pain	1	1	1.2	0	0	0.0	0	0	0.0
Nervous system disorders									
Headache	1	1	1.2	0	0	0.0	0	0	0.0
Peripheral sensory neuropathy	1	1	1.2	0	0	0.0	0	0	0.0
Renal disorders									
Acute kidney injury	1	1	1.2	0	0	0.0	0	0	0.0
Respiratory disorders									
Dyspnea	2	2	2.4	0	0	0.0	0	0	0.0
Cardiovascular disorders									
Hypertension	2	2	2.4	0	0	0.0	0	0	0.0

*N* number, *pts* patients, *γGT* gamma-glutamyl transferase, *LDH* lactate dehydrogenase.

## Discussion

Metastatic breast cancer still remains an incurable disease and the effective long-term management of patients represents a major challenge. Taxanes constitute a mainstay of treatment both in the early-stage, as well as in the advanced setting. However, the clinical use of these compounds is often associated with primary or acquired resistance. Consequently, the development of additional effective therapeutic options for patients previously treated with taxanes is important. So far, there are no standardised treatment regimens following the failure of anthracycline and taxane therapy. Capecitabine, gemcitabine, vinorelbine, eribulin, etc. are reasonable alternatives. Capecitabine has been the only approved treatment for anthracycline- and taxane-pretreated MBC for many years, since the majority of Phase 3 studies in this setting failed to demonstrate superior clinical outcomes with new agents, compared or combined with capecitabine. More recently, eribulin was approved for MBC progressing after chemotherapy regimens including anthracyclines and taxanes, following a Phase 3 trial, which showed significantly improved OS for eribulin in comparison with investigators’ treatment of choice.^13^

In the current multicentre, single-arm Phase 2 study, cabazitaxel showed promising activity as second-line treatment in taxane-pretreated patients with MBC. Among the 84 patients enrolled in the study, the ORR was 22.6%, while the disease-control rate was 53.6%. These results are considered encouraging taking into account that cabazitaxel was given in a pretreated patient population. Moreover, the median OS of 15.2 months is considered particularly promising, although the median PFS was 3.7 months. These findings are comparable with the results of phase 3 studies evaluating eribulin versus treatment of physician’s choice or capecitabine, in pretreated patients with MBC.^13,14^

Regarding the efficacy of cabazitaxel according to resistance to previous taxane treatment, no difference was demonstrated in ORR (23.3% and 20.5% in taxane-resistant and taxane-non-resistant cases, respectively). Moreover, the median time to objective response was also similar (64 days and 59.5 days, respectively). However, the duration of response was shorter in patients with taxane-resistant disease, compared with taxane-non-resistant cases (5.1 months vs. 8.6 months). Similarly, taxane-resistant patients had numerically shorter PFS (median 3.1 months vs. 4.3 months), as well as worse OS (median 12.7 months vs. 19.4 months) in comparison with those with taxane-non-resistant disease. Clinical data assessing the activity of cabazitaxel in patients with MBC is very limited. A Phase 2 study has evaluated the efficacy of cabazitaxel in 71 patients with taxane-resistant MBC.^10^ The ORR was 14%, whereas 25% of patients had stable disease. The median time to progression (TTP) was 2.7 months, while the median OS was 12.3 months. Moreover, another Phase 1/2 dose-escalating study assessed the combination of cabazitaxel with capecitabine in patients with MBC pretreated with anthracyclines and taxanes.^11^ In the 21 evaluable patients at the maximum tolerated dose, the ORR was 23.8%, and the median TTP was 4.9 months. The results of our study compare favourably with the findings in the above mentioned, previously published studies in pretreated patients with MBC. On the other hand, a randomised Phase 2 study comparing cabazitaxel versus weekly paclitaxel as neoadjuvant therapy in patients with early HER2-negative breast cancer demonstrated inferior activity of cabazitaxel with respect to the pathological complete response rate.^15^ The efficacy of cabazitaxel in other types of cancer has also been evaluated with encouraging results in recent studies.^16,17^ Regarding toxicity, cabazitaxel was tolerated fairly well in most of the patients. The incidence of grade 3–4 neutropenia was relatively high (22.6%), considering that primary prophylaxis with G-CSF was recommended in all patients. Recent studies evaluating cabazitaxel in pretreated patients with non-small-cell lung cancer and head and neck carcinoma, have also reported similar rates of severe (grade 3–4) neutropenia, despite primary prophylaxis with G-CSF.^16,17^ However, the rates of grade 3–4 neutropenia were considerably lower compared with the reported incidence in the pivotal study in prostate cancer.^9^ Nevertheless, in the pivotal study neutropenia was measured at the nadir in all patients and prophylactic G-CSF was not allowed at cycle 1. In general, severe neutropenic events were manageable in the majority of cases in our study. The safety profile of the study drug was similar to the known toxicity profile of cabazitaxel, no serious adverse events were assessed as unexpected and no new safety issues were revealed.

In conclusion, this Phase 2 trial suggests that cabazitaxel is active with a manageable toxicity profile when administered as second-line treatment in patients with HER2-negative MBC, who have received prior taxane therapy. These promising results support further evaluation of cabazitaxel in Phase 3 studies in patients with MBC.

## Data Availability

All data are available upon request.
